# Jamming Up the “β-Staple”: Regulation of SIRT1 Activity by Its C-Terminal Regulatory Segment (CTR)

**DOI:** 10.1016/j.jmb.2013.11.013

**Published:** 2013-11-19

**Authors:** Ruth Anne Pumroy, Gino Cingolani

**Affiliations:** Department of Biochemistry and Molecular Biology, Thomas Jefferson University, 233 South 10th Street, Philadelphia, PA 19107, USA

## Protein Acetylation: A Switch to Gene Expression

Acetylation is one of the most abundant post-translational modifications in biology and is conserved in all kingdoms of life. As many as 90% of proteins involved in *Salmonella enterica* metabolic pathways are acetylated [1], primarily at lysine residues, and more than 80% of human proteins are N-terminally acetylated [2]. Analogous to phosphorylation, attachment of an acetyl group to a protein amino acid is a reversible reaction catalyzed by dedicated enzymes that have acetyl-transferase or deacetylase activity. Perhaps the most widely studied and best documented example of acetylation affects histones, basic proteins tightly bound to DNA to form chromatin. Acetylation of critical lysines in histone tails decreases the overall positive charge, decreasing electrostatic interactions with DNA [3]. This is thought to promote chromatin decondensation and to enhance accessibility to RNA polymerase, thereby stimulating transcriptional activity. On the contrary, histone deacetylation is usually associated with a reduction of transcriptional activity. Thus, protein acetylation represents a versatile and reversible molecular switch of vital importance in biology.

## Sirtuins Are Ubiquitous NAD^+^-Dependent Deacetylases

There are four classes of histone deacetylases (HDAC) in eukaryotes, of which sirtuins (or class III) are highly conserved phylogenetically. One of the defining characteristics of sirtuins is the requirement of NAD^+^ as cofactor [4], a dependence that links sirtuin function to the metabolic state of the cell. Caloric restriction studies in yeast, worms and flies have shown increased longevity linked to the increased activity of homologs of the *Saccharomyces cerevisiae* founder sirtuin, Sir2 [4,5]. The human genome encodes seven sirtuins, named SIRT1–SIRT7 [5]. SIRT1 (also known as Sir2α) is the closest homolog of yeast Sir2, and as in this model organism, its activity is also increased due to caloric restriction [6,7]. SIRT1 has many important cellular targets, such as p53 [8], NF-κB [9], FOXO [10] and PPARγ [11], making it an attractive target for cancer therapeutics and longevity studies. At the structural level, all sirtuins have a conserved catalytic core composed of a Rossmann fold for NAD^+^ binding, a Zn^2+^ binding module and a helical module containing an NAD^+^-binding loop. NAD^+^ and the acetylated lysine substrate bind across this interface, with the acetylated ε-amino group of the lysine adjacent to the ribose moiety of NAD^+^, in a hydrophobic tunnel where catalysis occurs. The substrate is primarily bound by backbone interactions and the formation of a three-stranded antiparallel “β-staple”. Physiological acetyl-peptides are accommodated in SIRT1 active site mainly by hydrogen bonding with their main-chain backbone, allowing deacetylation without sequence conservation [12]. There appears to be no particular consensus sequence for substrate binding to sirtuins, but specificity is dependent on the amino acid context of the acetylated lysine [12,13]. After catalysis, the products of this reaction are 2′-*O*-acetyl-ADP-ribose (*O*AADPr), nicotinamide and the deacetylated substrate.

## Regulation of Human SIRT1 by Its C-Terminal Regulatory Segment

In addition to the conserved catalytic core, located from residue 230 to residue 500, SIRT1 also presents N- and C-terminal extensions found in no other human sirtuin, which are thought to play a regulatory function. Kang *et al.* identified a short stretch at the C-terminus of murine SIRT1 (residues 631–655) that is essential for deacetylase activity [14] and that competes with an endogenous inhibitory factor known as DBC1 (*D*eleted in *B*reast *C*ancer-*1*) for activation of SIRT1 catalytic core (SIRT1^CAT^) [15]. They proposed that SIRT1 C-terminal regulatory (CTR) region (SIRT1^CTR^) functions allosterically to alter the affinity of the catalytic core for the acetylated substrate. Concurrently, Pan *et al.* found that SIRT1^CAT^ has very low enzymatic activity on its own and that both the N-and C-term regions are able to enhance SIRT1^CAT^ activity [16]. They proposed that the N-terminal domain increases the rate of catalysis, while the C-terminal domain (residues 584–665) increases binding to NAD^+^ and can function in *trans*. In this issue of the *Journal of Molecular Biology*, Davenport *et al*. report two crystal structures of human SIRT1 catalytic domain bound to its CTR (residues 641–665), crystallized in the presence (*closed* state) and in the absence (*open* state) of cofactor NAD^+^ [17]. Unexpectedly, the authors identified a dramatic conformational change between the two states. Without NAD^+^ (which is reacted in crystal to generate adenosine diphosphatase ribose), SIRT1 adopts an open conformation with the Zn^2+^ binding and helical modules rotated with respect to the NAD^+^-binding domain. In the presence of cofactor, the SIRT1 helical module is tightly folded onto the catalytic domain, adopting a closed conformation. Davenport *et al.* showed that interaction between SIRT1^CAT^ and the CTR greatly stabilizes the catalytic core, which is quite unstable at 37 °C (the temperature at which most enzymatic studies are performed). This structural stabilization does not increase enzymatic activity but rather dampens SIRT1 activity *in vitro*. However, removing the last 12 residues of the CTR or disrupting a conserved salt bridge between residues R276 and E656 relieves this attenuation and increases SIRT1 activity. This begs the question as to whether the CTR regulates deacetylase activity only intramolecularly (in *cis*) or if a more complex intermolecular mechanism of *trans*-activation occurs in solution, as previously suggested [16]. The authors explore both alternatives, although a conclusive answer will require more extensive investigations *in vivo*. In solution, an excess of CTR can compete off bound CTR and form a complex with SIRT1^CAT^ that co-migrates on a gel-filtration column and the SIRT1^CAT^ and CTR were co-crystallized, suggesting a binding affinity at least in the micromolar range. However, an accurate dissociation constant between SIRT1^CAT^ and CTR could not be determined due to the tendency of SIRT1^CAT^ to aggregate in solution. To complicate the puzzle, the authors also identified a substrate-mimetic peptide projecting from C-terminal residues 504–510 of a crystallographic mate “stapled” inside SIRT1 active site. The backbone conformation adopted by this *pseudo*-substrate is superimposable to the p53 substrate peptide (HKK^Ac^LMF) previously analyzed in complex with an archaeal SIRT1 ortholog [18]. A unique leucine in the *pseudo*-substrate occupies the position of the substrate acetyl-lysine directly facing NAD^+^. This suggests that various regions in the regulatory domains could potentially occupy the substrate-binding pocket and auto-inhibit the enzyme, reconciling the observation that the isolated SIRT1^CAT^ is essentially inactive *in vitro* [16]. Although not directly shown in this paper, we speculate that binding of a CTR to SIRT1^CAT^ in the presence of N-terminal regulatory and CTR domains may contribute to removing a *pseudo*-substrate from the active site, stimulating catalytic activity.

## Perspectives and Future Directions

The studies of Davenport *et al.* provide structural evidence for a regulatory role of SIRT1 C-terminal domain on deacetylase activity. This includes not only the previously identified CTR but also the C-terminal *pseudo*-substrate spanning region 504–510 that occupies the cargo-binding groove. Building upon this work, it is foreseeable that at least two directions of research will be particularly interesting to explore. First, to delve into the exact regulatory role of SIRT1^CTR^, it is essential to study its properties in a physiological environment. Phosphorylation in the CTR [19] is likely to play a pivotal role in modulating the interplay between potential intramolecular auto-inhibition by *pseudo*-substrate moieties and availability of acetylated substrates and hence promote temporal and spatial control of deacetylase activity. Second, as elegantly suggested by this paper, the observation that a leucine side chain can functionally replace the side chain of an acetyl-lysine provides a powerful framework to engineer peptides (or peptide-mimetics) that could compete with acetyl-substrates and inhibit SIRT1 activity. Given the broad physiological importance of acetylation and the pivotal role of acetylation in turning genes on and off, potent and selective small molecules to modulate SIRT1 activity would be at the forefront of fighting cancer and metastatic proliferation.
